# Early Onset of Combined Oxidative Phosphorylation Deficiency in Two Chinese Brothers Caused by a Homozygous (Leu275Phe) Mutation in the *C1QBP* Gene

**DOI:** 10.3389/fped.2020.583047

**Published:** 2020-12-02

**Authors:** Jie Wang, Huan Li, Min Sun, Ying Yang, Qianli Yang, Bailing Liu, Fang Liu, Wen Hu, Yanmin Zhang

**Affiliations:** ^1^Shaanxi Institute for Pediatric Diseases, Xi'an Children's Hospital, Xi'an, China; ^2^Xi'an Key Laboratory of Children's Health and Diseases, Xi'an, China; ^3^Department of Cardiology of Xi'an Children's Hospital, Affiliated Children's Hospital of Xi'an Jiaotong University, Xi'an, China; ^4^Department of Ultrasound, Xijing Hospital, Fourth Military Medical University, Xi'an, China; ^5^Department of Ultrasound of Xi'an Children's Hospital, Affiliated Children's Hospital of Xi'an Jiaotong University, Xi'an, China; ^6^Department of Radiology of Xi'an Children's Hospital, Affiliated Children's Hospital of Xi'an Jiaotong University, Xi'an, China

**Keywords:** C1QPB, Leu275Phe, combined oxidative phosphorylation deficiency, mitochondrial cardiomyopathies, hypertrophic cardiomyopathy

## Abstract

Mitochondrial diseases constitute a group of heterogeneous hereditary diseases caused by impairments in mitochondrial oxidative phosphorylation and abnormal cellular energy metabolism. C1QBP plays an important role in mitochondrial homeostasis. In this study, clinical, laboratory examinations, 12-lead electrocardiographic, ultrasonic cardiogram, and magnetic resonance imaging data were collected from four members of a Chinese family. Whole exome were amplified and sequenced for the proband. The structure of protein encoded by the mutation was predicted using multiple software programs. The proband was a 14-year old boy with myocardial hypertrophy, exercise intolerance, ptosis, and increased lactate. His 9-year old brother exhibited similar clinical manifestations while the phenomenon of ptosis was not as noticeable as the proband. The onset of this disease was in infancy in both cases. They were born after uneventful pregnancies of five generation blood relative Chinese parents. A homozygous mutation (Leu275Phe) in the *C1QBP* gene was identified in both brothers in an autosomal recessive inherited pattern. Their parents were heterozygous mutation carriers without clinical manifestations. We demonstrated that a homozygous C1QBP- P.Leu275Phe mutation in an autosomal recessive inherited mode of inheritance caused early onset combined oxidative phosphorylation deficiency 33 (COXPD 33) (OMIM:617713) in two brothers from a Chinese family.

## Introduction

Mitochondrial disease is a type of inherited metabolic disorder caused by defects in mitochondrial metabolic enzymes that result in disorders pertaining to adenosine triphosphate (ATP) synthesis and in insufficient energy sources. Mitochondrial dysfunction primarily affects organs with high-energy requirements, such as the heart, brain, and muscles (1, 2). To-this-date, ~300 genes associated with mitochondrial disease have been identified (3). Mutations can directly affect oxidative phosphorylation (OXPHOS) subunits or indirectly impair OXPHOS activity by disrupting mitochondrial homeostasis (4). Primary deficiencies of the OXPHOS system have direct impacts on mitochondrial function and result in several disease phenotypes, such as mitochondrial cardiomyopathies, mitochondrial encephalomyopathies, and mitochondrial myopathies (5).

Complement component 1 Q subcomponent-binding protein (C1QBP), also known as p32, is an evolutionary conserved and ubiquitously expressed multifunctional protein (6). Additionally, it is a predominant mitochondrial matrix protein involved in inflammation and infection processes, mitochondrial ribosome biogenesis, regulation of apoptosis and nuclear transcription, and pre-messenger ribonucleic acid (mRNA) splicing (7–10). C1QBP dysfunction could lead to a reduction of OXPHOS enzymes and mitochondrial energy metabolism disorders that may be attributed to a severely impaired mitochondrial protein synthetic process (11). Up to now, only six cases were reported with the mutations in the C1QBP gene in an autosomal recessive pattern. Feichtinger et al. (12) reported four individuals with biallelic mutations in C1QBP. The biallelic mutation of C1QBP caused a combined oxidative phosphorylation deficiency 33 (COXPD 33) (OMIM:617713). COXPD 33 was associate with mitochondrial cardiomyopathy, has variable onset (including intrauterine or neonatal forms), phenotypes and severity. Marchet et al. (13) reported two unrelated adult patients from consanguineous families with homozygous mutations in C1QBP were reported. They presenting with progressive external ophthalmoplegia (PEO), mitochondrial myopathy and without any heart involvement.

Herein, we report a homozygous mutation in *C1QBP* caused COXPD 33 in two Chinese brothers. The brothers had an early onset COXPD 33 with clinical manifestations of hypertrophic cardiomyopathy (HCM), exercise intolerance, and documented patterns of increased lactate.

## Methods

### Patients and Clinical Investigation

**Figure 1:**
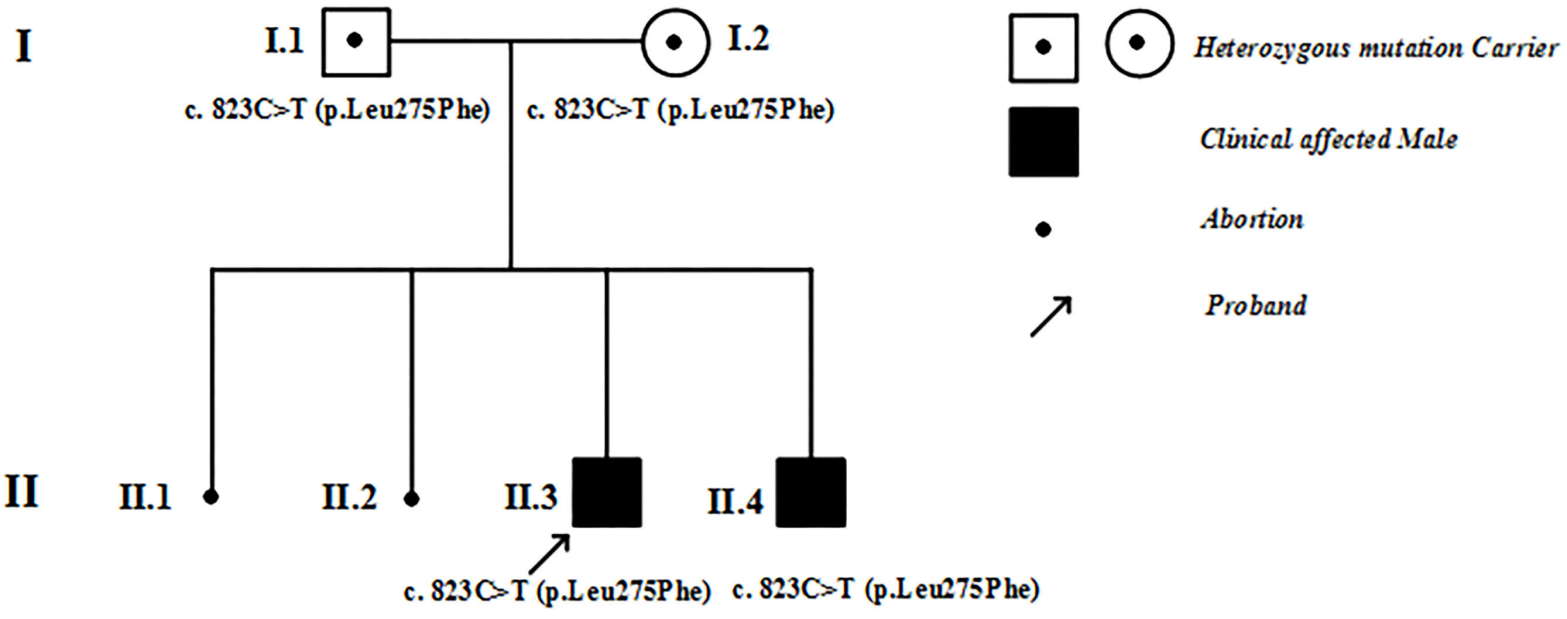
Family pedigree. I, II refer to the first and second generations in this family. Square symbols represent males, circles denote females. Filled black symbols represent patients with hypertrophic cardiomyopathy, and symbols with dots represent mutation carriers without clinical manifestations. The black spots denote abortions. The arrows indicate the proband of this family.

The clinical evaluation was conducted in accordance with the principles of the Declaration of Helsinki. The study was approved by the ethics committee of Xi'an Children's Hospital, the affiliated Children's Hospital of Xi'an Jiaotong University in China. Informed written consent was obtained from all the members of two generations in a Chinese family (Figure 1). In the cases of children with ages < 16 years, written informed consent was obtained from the parents. All evaluations included the medical history, family history, physical and laboratory examinations, 12-lead electrocardiographs (ECGs), ultrasonic cardiograms (UCGs), and magnetic resonance imaging (MRI) data.

According to the latest guidelines (14), the clinical diagnosis of HCM was established when the maximum left ventricular wall thickness (MLVWT) ≥ 15 mm in adults. For children with ages < 18 years, the diagnosis of HCM required a wall thickness ≥ 2 standard deviations (SD) above the predicted mean (Z-score ≥ 2) for age, gender, and body size.

### Genetic Analysis

Genomic DNA was extracted from 3 mL of whole blood with a blood genomic DNA Mini Kit (CW2087M, CWBIO, Beijing, China). Whole exome were captured (SureSelect Human All Exon V6, Agilent, USA) and sequenced on Illumina Nova Seq sequencing platform (Nova seq 6,000, Illumina, USA). Alignment and variant calling were performed with an information technology, platform-specific pipeline software (Torrent Suite, version 4.2) with the plug-in “variant caller” program (Life Technologies). Variants with (a) a minor allele frequency < 0.05 in population databases, such as 1,000 genome, ExAC, ESP, and GnomAD databases, or (b) those which were present in the Human Gene Mutation Database were included in the analysis. The identified mutation was verified among the remaining family members by Sanger sequencing. The copy number variation analysis was predicted based on whole exon next-generation sequencing data. Mitochondrial DNA was extracted from 3 mL of whole blood with a mitochondrial DNA isolation kit (K280-50, Biovision, America). The experiment of long-PCR amplification of mitochondrial DNA was performed in Fulgent Gene Biotechnology Co., Ltd. The American College of Medical Genetics and Genomics standards and guidelines were followed in this study for the interpretation of sequence variants (15).

### Structure Prediction of the Protein Encoded by the C1QBP- P.Leu275Phe Mutation

The three-dimensional (3D) structure of the protein encoded by C1QBP- P.Leu275Phe was analyzed using the wild type of C1QBP (PDB accession codes 1P32, https://www.rcsb.org/structure/1P32) and the SWISS-MODEL (http://swissmodel.expasy.org/). Images were acquired with the PyMOL molecular graphics system (PyMOL, https://pymol.org/2/).

## Result

### Clinical Characteristics

The proband was a 14-year old boy. Birth weight, length, and head circumference were within normal limits. His early milestone acquisitions were appropriate for his age. He presented with systemic edema and admitted to the local hospital when he was one and a half years. The UCG examination revealed left ventricular hypertrophy and ventricular endocardium (END) thickened with endocardial fibroelastosis (EFE) was diagnosed. After symptomatic treatment, the edema was reduced. He was then discharged with continuous oral administration of captopril 6.25 mg/24 h, digoxin 0.0625 mg/48 h, and prednisone 5 mg/48 h. Approximately 7 years later, he stopped captopril. Meanwhile, the dosage of digoxin was weaned from 0.0625 to 0.0417 mg/48 h and the dosage of prednisone was increased from 5 to 10 mg/48 h. The regular UCG examination showed that the thickness of left ventricular posterior wall (LVPW) and interventricular septum (IVS) were increased from 9 to 14 mm and 8 to 9 mm, respectively. Thickness of END was about 2.1–3.5 mm, without significant thicken compared with that of onset time. Furthermore, exercise intolerance with fatigue developed gradually. The patient was referred to the pediatric cardiology department of Xi'an Children's hospital at the age of 14. Physical examination revealed the boy is 132.5 cm tall and weighs 24.5 kg, slack skins, upturned nose, and ptosis (with an ~50% coverage of the cornea). He had a blood pressure of 100/58 mmHg with a regular pulse rate at 86 beats per min (bmp). He was afebrile at 36.7°C, and had a respiratory rate of 24 times/min. And breath sound was clear without wet and dry rales in both lungs. There was no bulge or tremor in the precordial region, and percussion heart boundary enlarged to the left. A regular heartbeat at 86 bpm, and heart sound was strong. No obvious murmur was detected. The abdomen was flat and soft, and there were no palpable enlargements of the liver, spleen, and there were no abnormalities of the nervous system.

The proband's brother, who is a 9-year-old boy. Birth weight, length, and head circumference were also within normal limits. He was admitted to a local hospital with pneumonia when he was 2 years old. The UCG examination showed that the thickness of END significantly increased to ~5.1 mm. He was also diagnosed with EFE. Diuretics and myocardial nutrients were administered. He also received orally captopril, digoxin, and prednisone at the same dosages as those administered to his brother. The UCG results showed that the thickness of the IVS and the LVPW endocardium gradually thickened from 7 to 9.7 mm, and from 9 to 13.7 mm, respectively. The thickness of the END appeared to recover from 5.1 to 3.9 mm compared with the initial recording. Gradually, he also developed exercise intolerance. He was referred to our hospital together with his brother. Physical examination showed that he had a blood pressure of 100/60 mmHg with a regular pulse rate at 90 bpm. He was afebrile at 36.6°C, and had a respiratory rate of 24 times/min. His lung breath sounds were clear, no bulge or tremor in the precordial region, and the lower left side of the heart is enlarged. A regular heartbeat at 90 beats per min, and heart sound was strong. No murmur was detected in each auscultatory valve areas. The abdomen was flat and soft. Additionally, there were no palpable enlargements of the liver, spleen, and there were no abnormalities found in the nervous system.

Figure 1 shows the pedigree of the family. The proband (II-3) and his brother (II-4) were born after uneventful pregnancies to Chinese parents. Their mother had two spontaneous abortions at 8 weeks of pregnancy before she gave birth to the proband.

**Figure 2:**
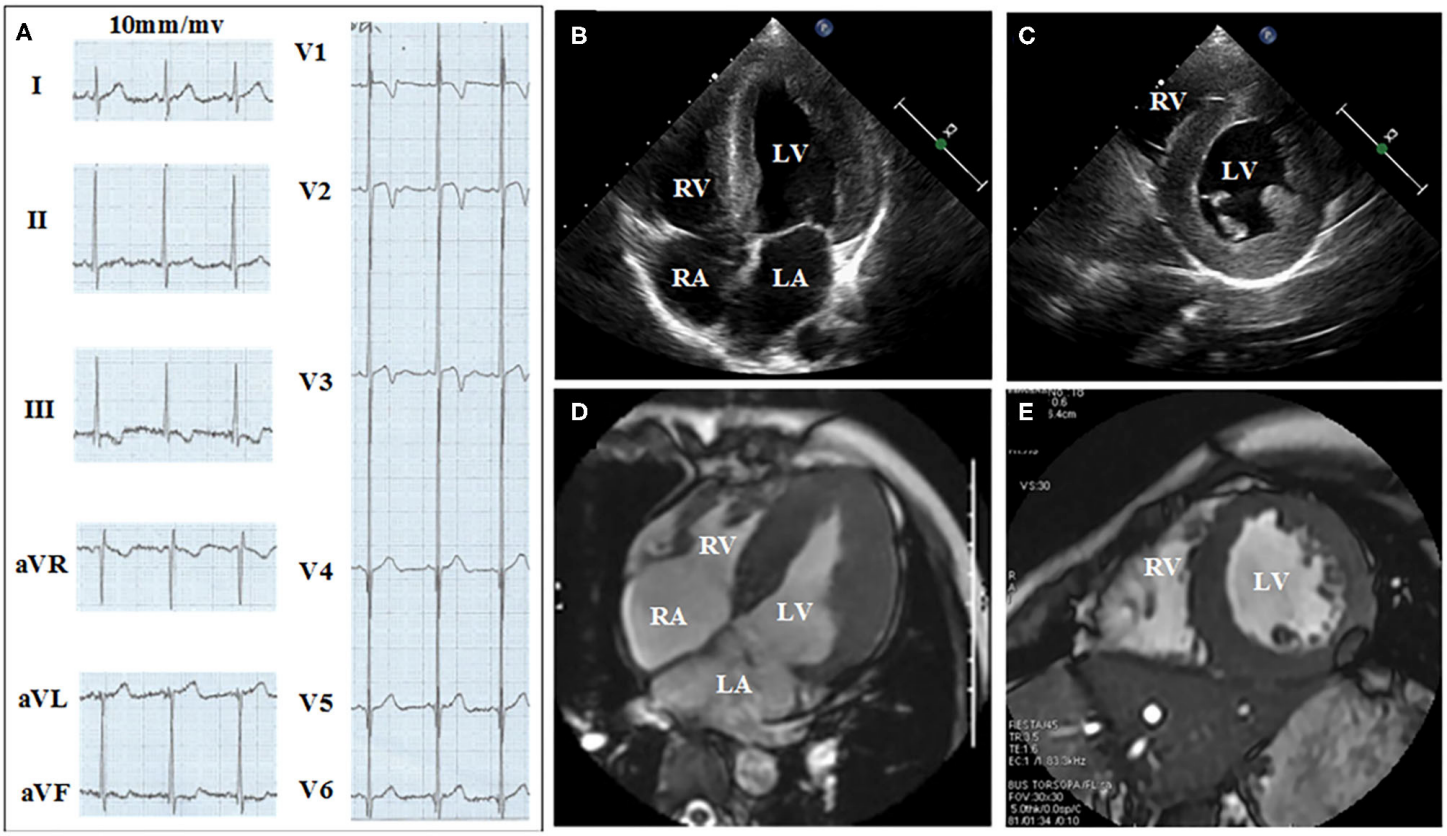
Electrocardiograph (ECG), ultrasonic cardiogram (UCG), and Magnetic Resonance Imaging (MRI) results acquired from the proband show the thickness of the septum and the left ventricular. **(A)** Twelve-lead ECG strips; UCG images of proband in **(B)** four-chamber and **(C)** short-axis views; MRI of proband in **(D)** four-chamber and **(E)** short axis views.

**Figure 3:**
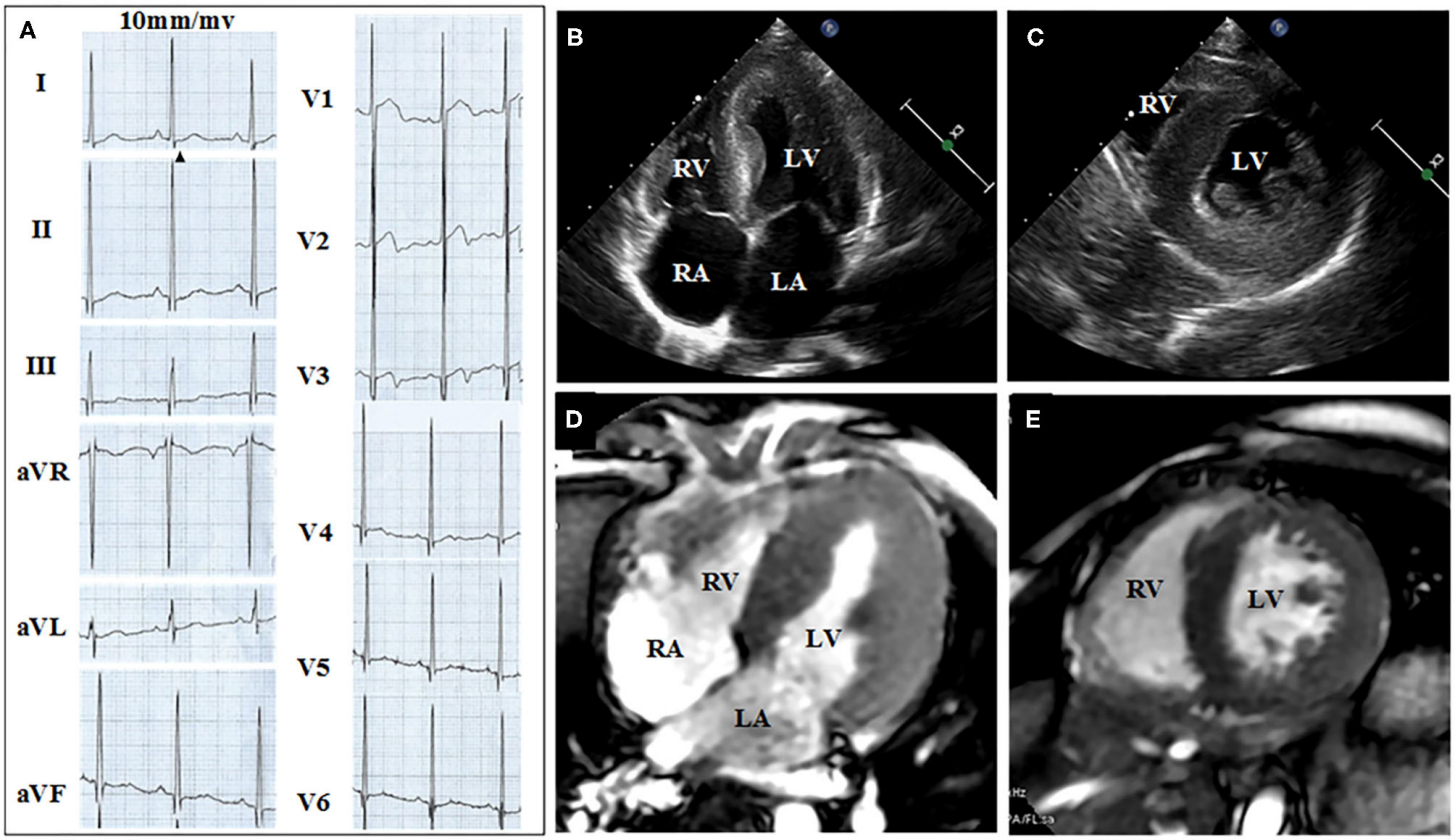
ECG, UCG, and MRI of the younger brother show the thickness of septal and the left ventricular wall. **(A)** Twelve-lead ECG strips; UCG images of brother in **(B)** four-chamber and **(C)** short-axis views; MRI of brother in **(D)** four-chamber and **(E)** short-axis views.

Figures 2, 3 show the results of ECG, UCG, and MRI. The ECG of the proband (Figure 2A) and his younger brother (Figure 3A) showed the voltages of QRS wave were increased significantly (leads V1–V6) that were indicative of cardiac hypertrophy. The UCG results of the proband (Figures 2B,C) showed that the thicknesses of LVPW, IVS, and END, were ~14, 9, and 2.1–3.5 mm, respectively. The UCG results of the younger brother (Figures 3B,C) show that the thicknesses of LVPW, IVS, and END, were 13.7, 9.7, and 1.9–3.9 mm, respectively. These findings were consistent with the MRI scan (Figures 2D,E for the proband, Figures 3D,E for the younger brother). Thus, the two brothers were diagnosed with left ventricle hypertrophy. And their parents were asymptomatic without reported syncope or cardiac arrest.

Table 1 summarizes the clinical characteristics and the laboratory test results of the two brothers. The blood lactate and ammonia in two brothers were higher than normal limits (Table 1). The concentrations of the N-terminal probrain natriuretic peptide (NT-proBNP) and high-sensitive cardiac troponin (hs-TnT) were increased in the two brothers, which suggesting a cardiac functional damage. The GC-MS analyses of organic acids in the urine showed that the values for lactic-2, 2-OH-isovaleric-2, 4-OH-phenyllactic (PHPLA)-3, 2-Keto-isovaleric-OX-2, 2-K o-isocaproic-OX-2, and 2-Keto-3-Methylvaleric-OX-2 were increased (Table 1). The remaining organic acids in the urine were normal (Supplementary Figure 1). The serum free fatty acid acyl-carnitine analysis using HPLC-QQQ-MS were normal (Supplementary Table 1). There were no significant abnormalities in liver function, blood glucose, and myocardial enzymes—including aspartate aminotransferase (AST), creatine kinase (CK), creatine kinase-MB (CK-MB), α-hydroxybutyrate dehydrogenase (α-HBDH), lactate dehydrogenase and its isoenzyme (LDH and LDH-1)—in these two brothers.

**Table 1:** Genetic and clinical findings in individuals with C1QBP mutation.

	**Proband (II-3)**	**Brother (II-4)**	
**Basic information**			
Gender	Male	Male	
Age of onset	1 and a half years old	2 years old	
Current age	14 years old	9 years old	
**Site of mutation**			
C1QBP variant (GenBank: NM_001212.3)	c. 823C>T (p. Leu275Phe)	c. 823C>T (p. Leu275Phe)	
**Physical examination**			
Height	132.5 cm	124.5 cm	
Weight	24.5 kg	23 kg	
Exercise tolerance	Decreased	Decreased	
Ptosis	Noticeable	Unapparent	
**Lab examinations**			
Blood lactate	2.58 mmol/L (0.7–2.1)	3.29 mmol/L (0.7–2.1)	
Blood ammonia	42.34 μmol/L (9–30)	20.92 μmol/L (9–30)	
Blood glucose	4.36 mmol/L (3.5–5.6)	4.72 mmol/L (3.5–5.6)	
NT-proBNP	871.30 pg/ml (<300)	428.80 pg/ml (<300)	
hs-TnT	14.97 pg/ml (0–14)	24.07 pg/ml (0–14)	
**Urine organic acid analysis**			
Lactic-2	66.80 (0.00–6.70)	452.48 (0.00–6.70)	
2-OH-isovaleric-2	6.62 (0.00–0.50)	19.18 (0.00–0.50)	
4-OH-phenyllactic (PHPLA)-3	114.15 (0.00–12.51)	313.75 (0.00–12.51)	
2-Keto-isovaleric-OX-2	1.16 (0.00–0.50)	3.44 (0.00–0.50)	
2-Keto-isocaproic-OX-2	2.90 (0.00–0.73)	8.94 (0.00–0.73)	
2-Keto-3-methylvaleric-OX-2	1.61 (0.00–0.50)	4.10 (0.00–0.50)	
**UCG**			
LVIDd	3.8 cm	3.5 cm	
LVIDs	2.6 cm	2.1 cm	
LA	22 mm	21 mm	
LA (Length/width)	27/18 mm	32/27 mm	
RA (Length/width)	30/29 mm	29/28 mm	
LV (Length/width)	57/31 mm	59/36 mm	
RV (Length/width)	49/30 mm	48/25 mm	
Thickness of END	2.1–3.5 mm	1.9–3.9 mm	
Thickness of IVS	9 mm	9.7 mm	
Thickness of LVPW	14 mm	13.7 mm	
EF	60%	68%	
**MRI**			
Short-Axis view	LVTDd	38.0 mm	31.2 mm
	LVTDs	15.4 mm	11.5 mm
	Thickness of LVWd	7.38 mm	7.2 mm
	Thickness of LVPWs	19.5 mm	20.9 mm
Four-Chamber view	LVLDd	67.8 mm	66.3 mm
	LVLDs	52.4 mm	50.4 mm
	Thickness of IVSd	14.1 mm	15.1 mm
	Thickness of IVSs	16.5 mm	21.1 mm
LVOT diameter	15.5 mm	13.0 mm	

*(s, end-systolic; d, end diastolic) LVID, left ventricular internal dimension; LAID, left atrium internal dimension; LA, left atrium; RA, right atrium; LV, left ventricular; RV, right ventricular; END: endocardium; IVS, interventricular septum; LVPW, left ventricular posterior wall; EF, ejection fraction; LVTD, left ventricular internal trans dimension; LVWd, Thickness of left ventricular wall; LVLW, Thickness of left ventricular lateral wall; LVLD, left ventricular long dimension; LVOT, left ventricular out flow tract*.

### Genetic and Bioinformatics Analysis

**Figure 4:**
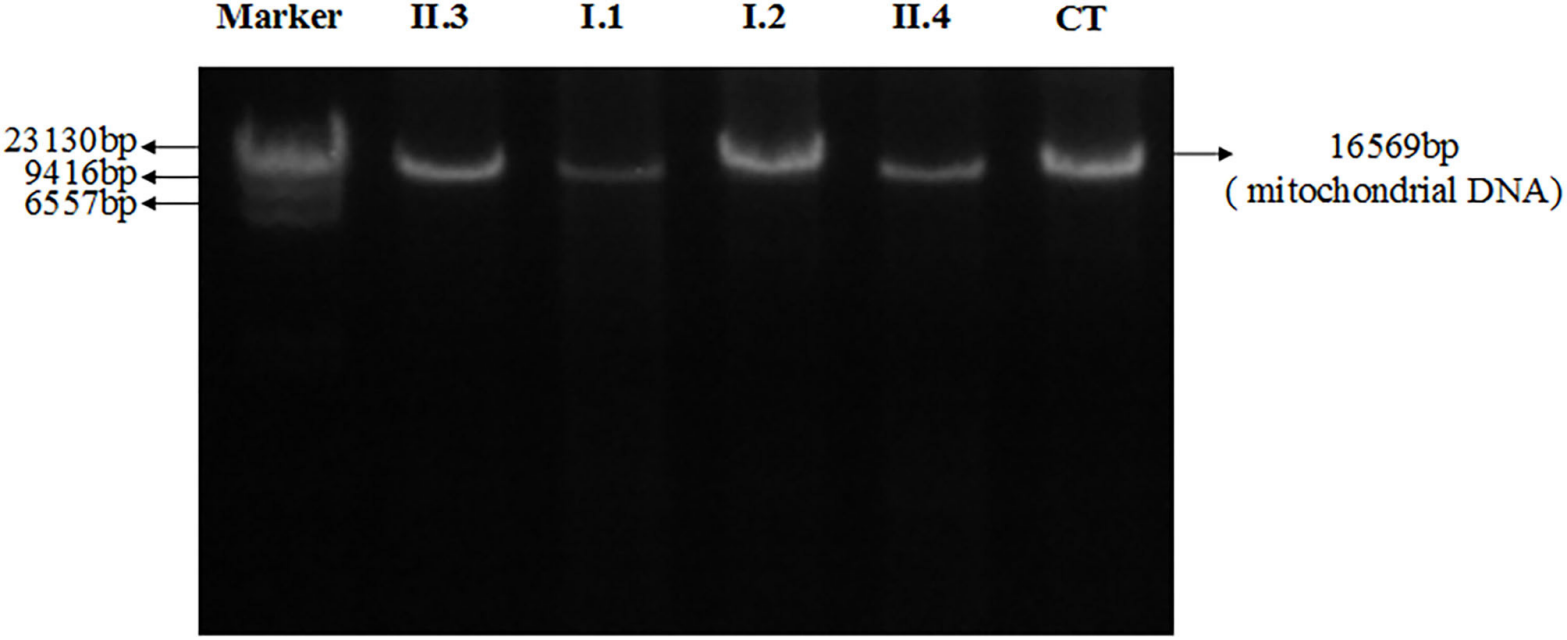
Electrophoresis results of amplified mitochondrial DNA by long-PCR of all patients and their parents. II.3: Proband; I.1: Father; I.2: Mother; II.4: Brother; CT: Control.

Figure 4 showed electrophoresis results of amplified mitochondrial DNA by long-PCR, and none large-scales deletion of mitochondrial DNA was detected in both patients and their parents.

**Figure 5:**
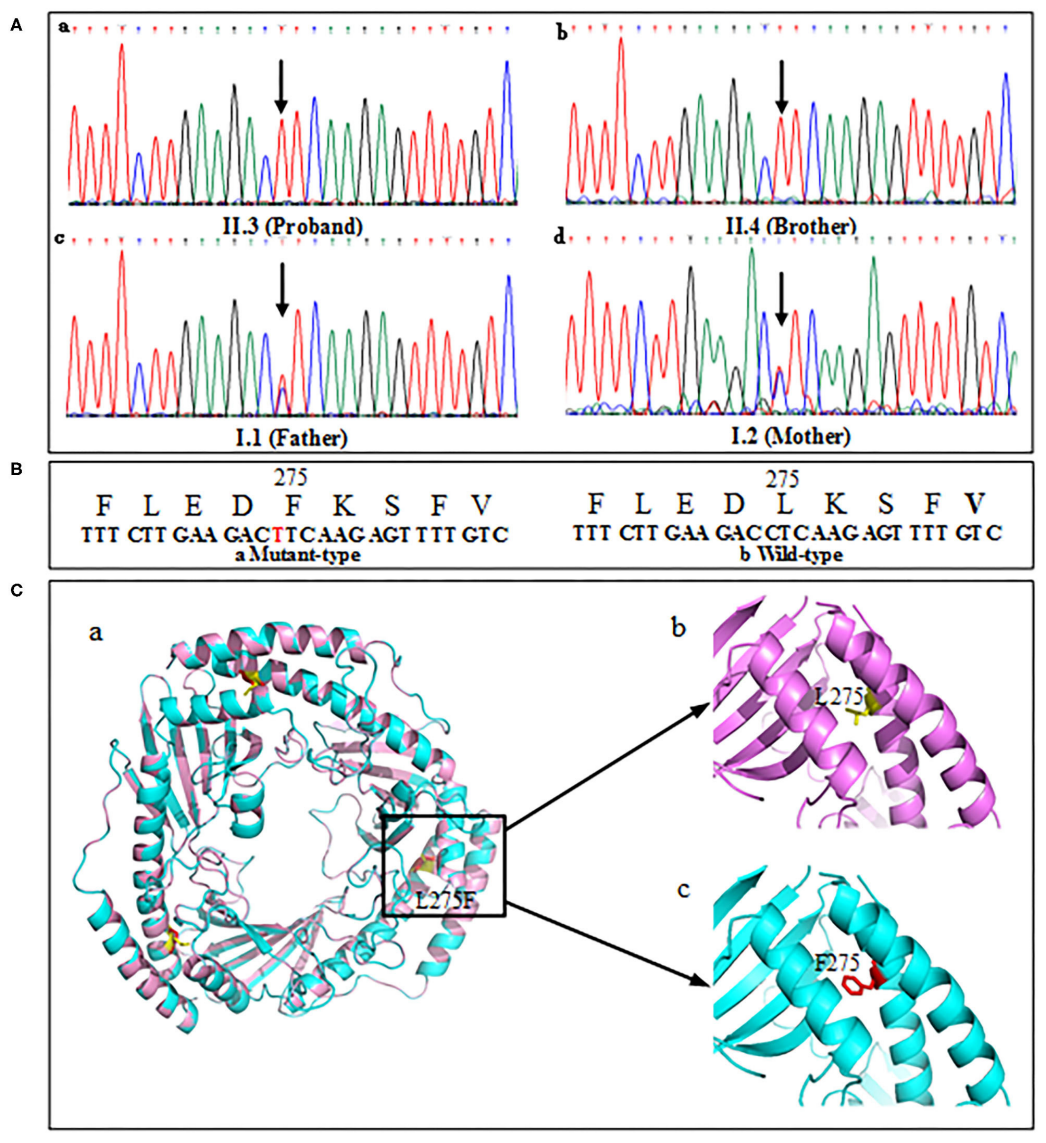
Mutation screening of tested family. **(A)** DNA changes identified based on sequencing. **(B)** Partial amino-acid sequence of wild-type (left) and mutant (right) were deduced. **(C,a)** Predicted three-dimensional structure of the C1QBP-L275F protein (PDB accession codes 1P32, https://www.rcsb.org/structure/1P32). **(C,b)** Wild type, L275 is shown in yellow color. **(C,c)** Mutation type, F275 is shown in red color.

The proband's genetic testing identified a homozygous mutation c.823C>T (Figure 5A,a) in exon 6 of the C1QBP gene (NM_001212.3) inherited from the mother (Figure 5A,c) and father (Figure 5A,d) in an autosomal recessive pattern. The parents were asymptomatic heterozygous mutation carriers. And the brother' mutation type (Figure 5A,b) was the same as proband. This mutation resulted in the substitution of leucine with phenylalanine at codon 275 (p. Leu275Phe) of the C1QBP protein (Figure 5B), denoted as C1QBP- p.Leu275Phe.

C1QBP- p.Leu275Phe has not been presented previously in 1,000 genome, ExAC, ESP and GnomAD databases. The pathogenicity of C1QBP- p.Leu275Phe was predicted to be damaging using multiple software programmes. Therefore, the variant of C1QBP- P.Leu275Phe are defined as likely pathogenic variants according to the ACMG guidelines. No abnormal copy number variations were found based on copy number variation analysis of whole exon next-generation sequencing data. And a variant list contains the genes that contained likely benign mutations and variants of unknown significance were showed in Supplementary Table 2.

Human C1QBP forms a homotrimer arranged in a doughnut-shaped structure with an unusually asymmetric charge distribution on the surface (8). There are three C1QBP molecules in an asymmetric unit that form a tightly bound trimer (Figure 5C,a). The visualization of the overall architecture shows that the β-sheets form a hyperboloid-shaped spool with the α-helices wrapped around it. Structural analyses of 3D model show that p. leu275 was localized in the αC helix of the protein (Figure 5C,b). The p. Phe275 of the mutation type is shown in Figure 5C,c.

## Discussion

In this study, we reported an early onset of COXPD 33 in two Chinese brothers with HCM, exercise intolerance, and increased lactate caused by the homozygous C1QBP- P.Leu275Phe mutation for the first time. The homozygous C1QBP- P.Leu275Phe mutation resulted in a defect in mitochondrial energy metabolism. HCM is one of the most common and important of cardiac phenotype associated with mitochondrial respiratory disorders (16, 17). Furthermore, the mitochondrial respiratory disorders is involves the development of heart failure (18, 19).

Prior research has indicated that a p32 mutation was the suspected cause of mitochondrial respiratory chain disorders (20). Four individuals from unrelated families with biallelic mutations in *C1QBP* had COXPD 33 (12). They were presented with exercise intolerance, progressive external ophthalmoplegia (PEO), and cardiomyopathy.

The onset age of the disease was variable. C1QBP- p.Gly247Trp and the p.Leu275Phe compound mutation caused intra-uterine neonatal cardiomyopathy. While the combinations of C1QBP-p.Cys186Ser and p.Pro204Leu mutation led to early death. The symptom onset of the homozygous mutation of C1QBP- p.Tyr188del was in adulthood. The only alive patient carried homozygous mutation of C1QBP- p.Leu275Phe. The diseases onset of this patient was in 5 years old with the increase of serum lactic acid, creatine kinase, transaminase, methionine and tyrosine. The clinical symptoms include left ventricular hypertrophy, fatiguability and ptosis. The primary clinical sign and symptoms were in line with our report. While the disease onset age in our study started from infancy which was earlier than that in the reported literature.

Feichtinger et al. (12) have also presented the functional studies muscular enzymology research. The functional studies of C1QBP- p.Leu275Phe mutation indicated that C1QBP protein could not be detected in the biopsy muscle of the patient, and C1QBP protein was significantly reduced in fibroblasts (primary fibroblast culture of the patient). Muscular enzymology studies showed a corresponding decrease in complex I and complex IV subunits, leading to mitochondrial respiratory chain defects.

Furthermore, two unrelated adult patients with homozygous mutations in C1QBP were reported (13). They presenting with progressive external ophthalmoplegia (PEO), mitochondrial myopathy and without any heart involvement. Muscle biopsies from both patients showed typical mitochondrial alterations and the presence of multiple mitochondrial DNA deletions. While in our study, the patients only have ptosis without any other eye problems. And none large-scales deletion of mitochondrial DNA was detected in both patients and their parents.

In addition, the mother of the proband in our study had two spontaneous abortions at 8 weeks of pregnancy before she gave birth to the proband. According to a prior report [11], C1QBP-deficient mice exhibited embryonic lethality. We suspect that the reason for the abortion was related to a C1QBP-deficiency. However, the exact cause of the spontaneous abortion could not be confirmed because autopsies were not performed in the cases of these two aborted fetuses.

The wild type (p. Leu275) and mutation type (p. Phe275) are localized in the αC helix of the C1QBP protein. The αC helix is an important structural domain of the protein. The N-terminal portion of the helix αC and Helix αB make extensive hydrophobic contacts with the β-sheet that are essential for the stability of the structure. Furthermore, the C-terminal portion of αC forms an antiparallel coiled-coil with the N-terminal helix αA. This coiled-coil region is important for protein–protein interactions and is responsible for homo-oligomerization (8). The amino acid residues of L and F are all nonpolar amino acids and have similar isoelectric points. This indicates that the properties of the amino acids do not influence the structure and function of C1QBP. The mutation C1QBP- P.Leu275Phe may affect the localization of the αC helix domain, and may thus increase the exposure of the hydrophobic surface. Furthermore, the coiled-coil regions of αA and αC were reported to form extensive intermolecular contacts (8). The mutation of the 275 amino acid residue in the αC helix domain may also influence the assembly of the C1QBP subunits. Thus, the preliminary conclusion is that the mutation C1QBP- P.Leu275Phe in the αC helix domain may lead to a faulty function of C1QBP owing to its effects on the localization of the αC helix domain, increase the exposure of the hydrophobic surface, or influence the assembly of subunits.

## Limitation

There are a few limitations in this study. First, we did not get the permission from the parents for muscle biopsy. Therefore, the C1QBP- p.Leu275Phe variant functional studies, muscular enzymology and histological/histochemical experiments were not performed. Secondly, the deep intronic mutations as well as complex indels deep intron variant could not been exclude using Next-generation sequencing.

## Conclusions

We demonstrated the clinical consequences of COXPD 33 caused by homozygous C1QBP- P.Leu275Phe mutations in autosomal recessive inherited mode in two Chinese brothers with early onset since infancy. The phenotypes were characterized by HCM, exercise intolerance, and increased lactate levels.

## Data Availability Statement

The original contributions presented in the study are included in the article/Supplementary Material, further inquiries can be directed to the corresponding author/s.

## Ethics Statement

Written informed consent was obtained from the individual(s), and minor(s)' legal guardian/next of kin, for the publication of any potentially identifiable images or data included in this article.

## Informed Consent

Informed written consent was obtained from all the members of two generations in a Chinese family. In the cases of children with ages < 16 years, written informed consent was obtained from the parents.

## Author Contributions

YZ: conceptualization. YZ, JW, HL, and MS: methodology. JW and QY: software. YY, BL, FL, and WH: validation. JW and YZ: formal analysis. JW, YZ, MS, and HL: writing original draft preparation. YZ, JW, HL, and MS: writing review and editing. All authors read and approved the final manuscript.

## Conflict of Interest

The authors declare that the research was conducted in the absence of any commercial or financial relationships that could be construed as a potential conflict of interest.
