# Supplementary material for: Early Onset of Combined Oxidative Phosphorylation Deficiency in Two Chinese Brothers Caused by a Homozygous (Leu275Phe) Mutation in the C1QBP Gene
Source: Front Pediatr. 2020 Dec 2;8:583047. doi: 10.3389/fped.2020.583047 (PMC7738465; doi:10.3389/fped.2020.583047)
Supplement: Supplementary file 1 [file Table_1.DOC]

Table SI. The screening diagnosis of inherited endocrine and metabolic diseases

| **No** | **Test Indexes** | **Proband** | **Brother** | **Reference Values** |
| --- | --- | --- | --- | --- |
|  |  | **II-3** | **II-4** | **/** |
| **The result of organic acids in urine** | | | | |
| 1 | Lactic-2 | 66.80 | 452.48 | 0.00～6.70 |
| 2 | 2-OH-isobutyric-2 | 0.56 | 0.71 | 0.00～0.50 |
| 3 | Hexanoic-1 | 0.04 | 0.09 | 0.00～0.50 |
| 4 | Glycolic-2 | 3.34 | 3.16 | 0.00～3.99 |
| 5 | Oxalic-2 | 3.95 | 0.00 | 0.00～1.00 |
| 6 | 2-OH-butyric-2 | 5.95 | 14.01 | 0.00～0.50 |
| 7 | Glyoxylic-OX-2 | 0.00 | 0.00 | 0.00～15.17 |
| 8 | 3-OH-propionic-2 | 2.32 | 3.38 | 0.00～1.95 |
| 9 | Pyruvic-OX-2 | 67.35 | 204.11 | 0.00～32.61 |
| 10 | Valproic(VPA)-1 | 0.32 | 0.91 | 0.00～0.50 |
| 11 | 3-OH-butyric-2 | 0.00 | 52.26 | 0.00～5.28 |
| 12 | 3-OH-isobutyric-2 | 15.54 | 0.00 | 0.00～13.02 |
| 13 | 2-OH-isovaleric-2 | 6.62 | 19.18 | 0.00～0.50 |
| 14 | 2-Methyl-3-OH-butyric-1-2 | 0.55 | 0.49 | 0.00～7.65 |
| 15 | Malonic-2 | 0.05 | 0.12 | 0.00～0.50 |
| 16 | 3-OH-isovaleric-2 | 0.66 | 0.74 | 0.00～6.10 |
| 17 | 2-Keto-isovaleric-OX-2 | 1.16 | 3.44 | 0.00～0.50 |
| 18 | Methylmalonic-2 | 0.22 | 0.04 | 0.20～5.34 |
| 19 | Ethylhydracrylic-2 | 6.16 | 7.84 | 0.00～6.22 |
| 20 | Urea-2 | 1.54 | 0.00 | 104.60~968.40 |
| 21 | 4-OH-butyric-2 | 0.00 | 0.00 | 0.00～0.50 |
| 22 | 2-OH-isocaproic-2 | 0.00 | 0.00 | 0.00～0.50 |
| 23 | 3-OH-valeric-2 | 0.00 | 0.00 | 0.00～0.50 |
| 24 | Acetoacetic | 0.13 | 0.67 | 0.00～0.10 |
| 25 | 2-OH-3-methylvaleric-2 | 0.13 | 0.26 | 0.00～0.50 |
| 26 | Benzoic-1 | 0.06 | 0.08 | 0.00～29.13 |
| 27 | Acetoacetic-OX-2 | 1.61 | 4.44 | 0.00～0.10 |
| 28 | Octanoic-1 | 0.00 | 0.00 | 0.00～0.59 |
| 29 | 2-Keto-3-methylvaleric-OX-2 | 1.61 | 4.10 | 0.00～0.50 |
| 30 | 2-Methyl-3-OH-valeric-2(1) | 0.00 | 0.00 | 0.00～0.50 |
| 31 | Glycerol-3 | 0.00 | 0.00 | 0.00～2.72 |
| 32 | Phosphoric-3 | 26.33 | 76.47 | 0.00～72.70 |
| 33 | 2-Methyl-3-OH-valeric-2(2) | 0.00 | 0.00 | 0.00～0.50 |
| 34 | Ethylmalonic-2 | 0.43 | 0.74 | 0.00～7.45 |
| 35 | 2-Keto-isocaproic-OX-2 | 2.90 | 8.94 | 0.00～0.73 |
| 36 | Acetylglycine-1 | 0.00 | 0.00 | 0.00～0.50 |
| 37 | Phenylacetic-1 | 0.00 | 0.00 | 0.00～0.62 |
| 38 | Maleic-2 | 0.19 | 0.19 | 0.00～0.55 |
| 39 | Succinic-2 | 0.64 | 0.80 | 6.50～129.90 |
| 40 | Methylsuccinic-2 | 0.53 | 0.74 | 0.00～8.62 |

**Continued table 1**

| **No** | **Test Indexes** | **Proband** | **Brother** | **Reference Values** |
| --- | --- | --- | --- | --- |
|  |  | **II-3** | **II-4** | **/** |
| 41 | Glyceric-3 | 0.26 | 0.36 | 0.00～2.93 |
| 42 | Uracil-2 | 0.00 | 0.00 | 0.00～19.65 |
| 43 | Fumaric-2 | 0.00 | 0.00 | 0.00～10.36 |
| 44 | Propionylglycine-1 | 0.00 | 0.00 | 0.00～0.50 |
| 45 | Acetylglycine-1 | 0.00 | 0.00 | 0.00～0.50 |
| 46 | Mevalonolactone-2 | 0.00 | 0.00 | 0.00～0.50 |
| 47 | Mevalonolactone-1 | 0.12 | 0.17 | 0.00～0.50 |
| 48 | Isobutyrylglycine-1 | 0.00 | 0.00 | 0.00～0.50 |
| 49 | 2-Propyl-3-OH-pentanoic(VPA)-2 | 0.00 | 0.00 | 0.00～0.50 |
| 50 | Mesaconic(Methylfumaric)-2 | 0.38 | 0.58 | 0.00～11.36 |
| 51 | Glutaric-2 | 0.39 | 0.40 | 0.00～8.44 |
| 52 | 3-Methylglutaconic-2 | 0.00 | 0.00 | 0.00～0.50 |
| 53 | 3-Methylglutaric-2 | 0.61 | 0.84 | 0.00～10.48 |
| 54 | 2-Propyl-3-ketopentanoic(VPA)-2 | 0.00 | 0.00 | 0.00～0.50 |
| 55 | Propionylglycine-2 | 0.00 | 0.00 | 0.00～0.50 |
| 56 | Isobutyrylglycine-2 | 0.00 | 0.00 | 0.00～0.50 |
| 57 | 2-Deoxytetronic | 0.30 | 0.95 | 0.00～12.88 |
| 58 | Butyrylglycine-1 | 0.07 | 0.26 | 0.00～0.50 |
| 59 | 3-Methylglutaconic-2 | 1.31 | 1.72 | 0.00～5.12 |
| 60 | Glutaconic-2 | 0.00 | 0.00 | 0.00～0.50 |
| 61 | Succinylacetone-OX-2(1) | 0.00 | 0.00 | 0.00～0.50 |
| 62 | Decanoic-1 | 0.00 | 0.00 | 0.00～0.50 |
| 63 | 2-Propyl-5-OH-pentanoic(VPA)-2 | 0.09 | 0.08 | 0.00～0.50 |
| 64 | 3-Methylglutaconic-2 | 0.50 | 0.75 | 0.00～14.15 |
| 65 | Isovalerylglycine-1 | 0.30 | 0.40 | 0.00～0.69 |
| 66 | Butyrylglycine-2 | 0.07 | 0.17 | 0.00～0.60 |
| 67 | Malic-3 | 0.00 | 0.00 | 0.00～1.20 |
| 68 | Adipic-2 | 0.00 | 0.00 | 0.50～13.00 |
| 69 | Isovalerylglycine-2 | 0.28 | 0.09 | 0.00～0.50 |
| 70 | 2-Hexenedioic-2 | 1.85 | 0.53 | 0.00～19.55 |
| 71 | 5-Oxoproline-2(pyroglutamic) | 0.25 | 0.14 | 0.00～10.48 |
| 72 | 3-methyladipic | 0.38 | 0.42 | 0.00～31.21 |
| 73 | Thiodiglycolic-2 | 0.44 | 0.67 | 0.00～0.10 |
| 74 | 2-Propyl-hydroxyglutaric(VPA)-2 | 0.00 | 0.00 | 0.00～0.10 |
| 75 | 7-OH-octanoic-2 | 0.00 | 0.00 | 0.00～0.06 |
| 76 | 5-OH-methyl-2-furoic-1 | 4.77 | 5.98 | 0.00～9.02 |
| 77 | Tiglylglycine-2 | 0.00 | 0.00 | 0.00～0.50 |
| 78 | 3-Methylcrotonylglycine-1 | 0.00 | 0.00 | 0.00～1.05 |
| 79 | Tiglylglycine-1 | 0.51 | 0.40 | 0.00～0.53 |
| 80 | 3-Methylcrotonylglycine-2 | 0.00 | 0.00 | 0.00～0.52 |
| 81 | 2-OH-glutaric-3 | 0.00 | 0.00 | 0.60～9.49 |

**Continued table 2**

| **No** | **Test Indexes** | **Proband** | **Brother** | **Reference Values** |
| --- | --- | --- | --- | --- |
|  |  | **II-3** | **II-4** | **/** |
| 82 | 3-OH-glutaric-3 Cf#81 | 2.07 | 3.27 | 0.00～0.50 |
| 83 | Phenyllactic | 0.00 | 0.00 | 0.00～5.79 |
| 84 | Pimelic-2 | 3.46 | 6.31 | 0.00～15.16 |
| 85 | 3-OH-3-methylglutaric-3 | 0.16 | 0.25 | 0.00～30.52 |
| 86 | 3-OH-phenylacetic-2 | 0.86 | 1.03 | 0.00～1.94 |
| 87 | 2-Ketoglutaric-OX-2(1) | 9.39 | 15.19 | 3.00～169.10 |
| 88 | 4-OH-benzoic-2 | 0.00 | 0.00 | 0.00～22.38 |
| 89 | 4-OH-phenylacetic | 8.84 | 9.98 | 8.60～139.99 |
| 90 | 2-Ketoglutaric-OX-2(2) | 0.08 | 0.09 | 0.00～28.54 |
| 91 | Hexanoylglycine-1 | 0.00 | 0.00 | 0.00～0.50 |
| 92 | Phenylpyruvic-OX-2 | 0.00 | 0.00 | 0.00～0.50 |
| 93 | N-Acetylaspartic-2 | 0.08 | 0.15 | 0.00～7.84 |
| 94 | 2-OH-adipic-3 | 0.26 | 0.79 | 0.00～3.34 |
| 95 | Octenedioic-2 | 0.57 | 0.66 | 0.00～8.37 |
| 96 | 3-OH-adipic-3 | 0.00 | 0.14 | 0.00～16.34 |
| 97 | Suberic-2 | 0.00 | 0.00 | 0.00～11.51 |
| 98 | 3-Methylglutaconic-2 | 0.00 | 0.00 | 0.00～0.50 |
| 99 | 2-Keto-adipic-OX-3 | 0.50 | 1.18 | 0.00～11.43 |
| 100 | Aconitic-3 | 29.68 | 48.55 | 15.10～304.07 |
| 101 | Orotic-3 | 0.00 | 0.00 | 0.00～2.50 |
| 102 | Vanillic-2 | 0.34 | 0.25 | 0.00～5.79 |
| 103 | Homovanillic-2(HVA) | 4.57 | 5.23 | 5.80～42.35 |
| 104 | Azelaic-2 | 3.00 | 1.50 | 0.00～21.63 |
| 105 | Hipupuric-2 | 0.00 | 0.00 | 0.00～21.54 |
| 106 | Isocitric-4 | 6.87 | 8.81 | 8.30～62.91 |
| 107 | Citric-4 | 13.53 | 15.66 | 31.40～978.29 |
| 108 | Homogentisic-3 | 0.00 | 0.00 | 0.00～0.99 |
| 109 | Hippuric-1 | 0.00 | 0.00 | 0.00～336.99 |
| 110 | Methylcitric-4(1) | 0.00 | 0.00 | 0.00～1.81 |
| 111 | 3-(3-OH-phenyl)-3-OH-propionic-3 | 5.53 | 4.75 | 0.00～4.99 |
| 112 | Methylcitric-4(2) | 0.00 | 0.00 | 0.00～1.49 |
| 113 | 3-OH-octenedioic-3 | 4.17 | 7.33 | 0.00～11.68 |
| 114 | 3-OH-suberic-3 | 2.27 | 3.93 | 0.00～8.95 |
| 115 | Vanilmandelic-3(VMA) | 16.22 | 17.95 | 11.70～84.60 |
| 116 | Sebacic-2 | 0.00 | 0.00 | 0.00～11.52 |
| 117 | Decadienedionic-2 | 0.11 | 0.20 | 0.00～4.64 |
| 118 | 4-OH-phenyllactic(PHPLA)-2 | 114.15 | 313.75 | 0.00～12.51 |
| 119 | 4-OH-phenylpyruvic(PHPPA)-OX-2 | 0.00 | 0.00 | 0.00～1.90 |
| 120 | 2-OH-hippuric-3 | 0.00 | 0.00 | 0.00～20.66 |
| 121 | Indole-3-acetic-2 | 0.57 | 0.24 | 0.00～149.51 |
| 122 | Suberylglycine-2 | 0.00 | 0.00 | 0.00～0.50 |

**Continued table 3**

| **No** | **Test Indexes** | **Proband** | **Brother** | **Reference Values** |
| --- | --- | --- | --- | --- |
|  |  | **II-3** | **II-4** | **/** |
| 123 | Palmitic-1 | 30.93 | 0.00 | 0.00～23.34 |
| 124 | 2-OH-sebacic-3 | 0.81 | 0.93 | 0.00～10.34 |
| 125 | 3-OH-sebacic-3 | 0.13 | 0.20 | 0.00～15.60 |
| 126 | 2-OH-hippuric-2 | 0.00 | 0.00 | 0.00～20.66 |
| 127 | Dodecanedioic-2 | 0.00 | 0.00 | 0.00～0.50 |
| 128 | N-Acetyltyrosine-3 | 0.00 | 0.00 | 0.00～0.10 |
| 129 | Uric-4 | 0.00 | 0.00 | 0.00～21.49 |
| 130 | 3,6-Epoxydodecanedioic-2 | 0.33 | 0.58 | 0.00～8.58 |
| 131 | 3-OH-dodecanedioic-3 | 0.00 | 0.00 | 0.00～2.25 |
| 132 | 3,6-Epoxytetradecanedioic-2 | 0.00 | 0.00 | 0.00～6.17 |
| **The result of amino acid and carnitine in serum (μM)** | | | | |
| 1 | Ala | 222.795 | 236.381 | 60.000~300.000 |
| 2 | Asp | 26.580 | 26.187 | 10.000～80.000 |
| 3 | Glu | 63.041 | 52.689 | 45.000~200.000 |
| 4 | Met | 13.017 | 15.000 | 8.000～35.000 |
| 5 | Phe | 44.569 | 50.383 | 20.000~120.000 |
| 6 | Tyr | 32.076 | 42.204 | 20.000~100.000 |
| 7 | Leu | 105.788 | 117.464 | 50.000~250.000 |
| 8 | Trp | 27.219 | 26.162 | 10.000～75.000 |
| 9 | Val | 155.208 | 170.762 | 80.000~300.000 |
| 10 | Arg | 6.890 | 9.061 | 1.500～25.000 |
| 11 | Cit | 19.738 | 20.104 | 7.000～35.000 |
| 12 | Gly | 173.279 | 154.971 | 90.000~350.000 |
| 13 | Orn | 17.097 | 20.826 | 15.000～80.000 |
| 14 | Gln | 6.926 | 8.355 | 6.000～30.000 |
| 15 | His | 79.578 | 115.248 | 10.000~300.000 |
| 16 | Ser | 56.430 | 44.502 | 20.000~100.000 |
| 17 | Thr | 27.164 | 31.492 | 15.000~100.000 |
| 18 | Pro | 164.839 | 147.231 | 90.000~1700.000 |
| 19 | Arg/Orn | 0.403 | 0.435 | 0.050～0.700 |
| 20 | Cit/Arg | 2.865 | 2.219 | 0.600～10 |
| 21 | Orn/Cit | 0.865 | 1.036 | 0.800～4.000 |
| 22 | Met/Phe | 0.312 | 0.298 | 0.200～0.600 |
| 23 | Leu/Phe | 2.374 | 2.331 | 1.500～4.000 |
| 24 | Phe/Tyr | 1.389 | 1.194 | 0.500～2.000 |
| 25 | Gly/Phe | 3.888 | 3.076 | 1.500～10.000 |
| 26 | Tyr/Phe | 0.720 | 0.838 | 0.500～2.000 |
| 27 | Glu/Cit | 3.194 | 2.621 | 2.450～15.000 |
| 28 | His/Phe | 1.785 | 2.287 | 0.000～4.000 |
| 29 | Thr/Phe | 0.609 | 0.625 | 0.000～1.500 |
| 30 | Trp/Phe | 0.611 | 0.519 | 0.200～1.200 |

**Continued table** 4

| **No** | **Test Indexes** | **Proband** | **Brother** | **Reference Values** |
| --- | --- | --- | --- | --- |
|  |  | **VI-3** | **VI-4** | **/** |
| 31 | Cit/Phe | 0.443 | 0.399 | 0.150～0.800 |
| 32 | Glu/Phe | 1.414 | 1.046 | 1.000～5.000 |
| 33 | C0 | 11.710 | 13.729 | 10.000～60.000 |
| 34 | C2 | 5.915 | 5.434 | 6.000～30.000 |
| 35 | C3 | 0.963 | 0.876 | 0.500～4.000 |
| 36 | C3DC | 0.161 | 0.262 | 0.030～0.400 |
| 37 | C4 | 0.192 | 0.107 | 0.060～0.500 |
| 38 | C4-OH | 0.103 | 0.066 | 0.020～0.350 |
| 39 | C4DC | 0.328 | 0.323 | 0.200～1.200 |
| 40 | C5 | 0.061 | 0.081 | 0.040～0.300 |
| 41 | C5:1 | 0.008 | 0.018 | 0.000～0.100 |
| 42 | C5-0H | 0.178 | 0.203 | 0.060～0.600 |
| 43 | C5DC | 0.172 | 0.119 | 0.000～0.200 |
| 44 | C6 | 0.038 | 0.055 | 0.010～0.150 |
| 45 | C6:1 | 0.020 | 0.035 | 0.010～0.100 |
| 46 | C6-OH | 0.019 | 0.020 | 0.000～0.100 |
| 47 | C6DC) | 0.022 | 0.017 | 0.000～0.060 |
| 48 | C8) | 0.048 | 0.088 | 0.010～0.300 |
| 49 | C8:1 | 0.094 | 0.119 | 0.030～0.500 |
| 50 | C8:2 | 0.015 | 0.000 | 0.000～0.100 |
| 51 | C8DC | 0.013 | 0.010 | 0.000～0.040 |
| 52 | C10 | 0.053 | 0.121 | 0.020～0.500 |
| 53 | C10:1 | 0.115 | 0.184 | 0.030～0.450 |
| 54 | C10:2 | 0.029 | 0.040 | 0.000～0.100 |
| 55 | C10DC | 0.370 | 0.356 | 0.020～0.400 |
| 56 | C12 | 0.037 | 0.037 | 0.020～0.200 |
| 57 | C12:1 | 0.047 | 0.062 | 0.020～0.200 |
| 58 | C12:2 | 0.017 | 0.020 | 0.000～0.100 |
| 59 | C12-OH | 0.018 | 0.022 | 0.000～0.100 |
| 60 | C12DC | 0.013 | 0.014 | 0.000～0.150 |
| 61 | C14 | 0.030 | 0.024 | 0.020～0.250 |
| 62 | C14:1 | 0.055 | 0.037 | 0.010～0.300 |
| 63 | C14:2 | 0.037 | 0.037 | 0.000～0.200 |
| 64 | C14-OH | 0.030 | 0.024 | 0.000～0.060 |
| 65 | C14DC | 0.018 | 0.020 | 0.000～0.150 |
| 66 | C16 | 0.495 | 0.461 | 0.300～2.000 |
| 67 | C16:1 | 0.034 | 0.067 | 0.020～0.200 |
| 68 | C16:2 | 0.008 | 0.017 | 0.000～0.050 |
| 69 | C16-OH | 0.008 | 0.035 | 0.000～0.050 |
| 70 | C16:1-OH | 0.015 | 0.043 | 0.000～0.120 |
| 71 | C16DC | 0.009 | 0.011 | 0.000～0.030 |

**Continued table 5**

| **No** | **Test Indexes** | **Proband** | **Brother** | **Reference Values** |
| --- | --- | --- | --- | --- |
|  |  | **VI-3** | **VI-4** | **/** |
| 72 | C18 | 0.360 | 0.281 | 0.200～1.200 |
| 73 | C18:1 | 0.423 | 0.392 | 0.300～1.800 |
| 74 | C18:2 | 0.227 | 0.244 | 0.050～0.600 |
| 75 | C18-OH | 0.008 | 0.009 | 0.000～0.030 |
| 76 | C18:1-OH | 0.019 | 0.014 | 0.000～0.100 |
| 77 | C18DC | 0.014 | 0.011 | 0.000～0.030 |
| 78 | C3/C2 | 0.163 | 0.161 | 0.040～0.250 |
| 79 | C3DC/C4 | 0.835 | 2.437 | 0.200～2.000 |
| 80 | C4/C2 | 0.033 | 0.020 | 0.000～0.050 |
| 81 | C4/C3 | 0.200 | 0.123 | 0.040～0.400 |
| 82 | C4-OH/C2 | 0.017 | 0.012 | 0.000～0.020 |
| 83 | C4-OH/C3 | 0.107 | 0.075 | 0.000～0.300 |
| 84 | C5/C2 | 0.101 | 0.015 | 0.000～0.020 |
| 85 | C5/C3 | 0.063 | 0.093 | 0.000～0.200 |
| 86 | C5-OH/C3 | 0.185 | 0.232 | 0.040～0.400 |
| 87 | C5-OH/C8 | 3.685 | 2.315 | 0.500～10.000 |
| 88 | C5DC/C3 | 0.179 | 0.136 | 0.000～0.200 |
| 89 | C5DC/C8 | 3.563 | 1.357 | 0.100～2.500 |
| 90 | C5DC/C16 | 0.347 | 0.258 | 0.010～0.150 |
| 91 | C6/C3 | 0.040 | 0.062 | 0.010～0.150 |
| 92 | C8/C3 | 0.050 | 0.100 | 0.010～0.250 |
| 93 | C8/C10 | 0.909 | 0.724 | 0.400～1.500 |
| 94 | C10/C3 | 0.055 | 0.138 | 0.010～0.350 |
| 95 | C12/C3 | 0.038 | 0.042 | 0.000～0.200 |
| 96 | C14/C3 | 0.031 | 0.028 | 0.000～0.200 |
| 97 | C14:1/C8:1 | 0.586 | 0.308 | 0.070～2.000 |
| 98 | C14:1/C16 | 0.111 | 0.079 | 0.020～0.350 |
| 99 | C16/C2 | 0.084 | 0.085 | 0.030～0.150 |
| 100 | C16/C3 | 0.514 | 0.527 | 0.200～1.500 |
| 101 | C18/C3 | 0.374 | 0.321 | 0.100～1.000 |
| 102 | C14-OH/C3 | 0.031 | 0.028 | 0.000～0.060 |
| 103 | C16-OH/C3 | 0.008 | 0.039 | 0.000～0.040 |
| 104 | C18-OH/C3 | 0.008 | 0.010 | 0.000～0.030 |
| 105 | (C16+C18:1)/C2 | 0.155 | 0.157 | 0.060～0.250 |
| 106 | C0/(C16+C18) | 13.699 | 18.491 | 10.000～40.000 |
